# Advances in the study of selenium and human intestinal bacteria

**DOI:** 10.3389/fnut.2022.1059358

**Published:** 2022-12-14

**Authors:** Jinzhong Cai, Weizhu Su, Xianxian Chen, Heng Zheng

**Affiliations:** ^1^School of Chemistry, Chemical Engineering and Life Sciences, Wuhan University of Technology, Wuhan, China; ^2^Department of Interventional Radiology, Shenzhen People's Hospital (The First Affiliated Hospital, Southern University of Science and Technology), Shenzhen, China; ^3^Dental Department, Shenzhen People's Hospital (The First Affiliated Hospital, Southern University of Science and Technology), Shenzhen, China; ^4^School of Resources and Environmental Engineering, Wuhan University of Technology, Wuhan, China

**Keywords:** selenium, human health, intestinal bacteria, microflora, disease

## Abstract

Selenium (Se) is an essential trace element for humans and has conveyed great a wide range of interests due to its contribution to health. Presently, the regulatory mechanisms of selenium on human health, especially the regulatory mechanisms of selenium on human intestinal (gut) microflora and its effects on diseases are receiving attention from academic circles. This review involves the effects of selenium on physical health, the relationship between selenium and intestinal microflora, and the progress of research between selenium, intestinal microflora, and diseases. Furthermore, the current status of research on the selenium, intestinal microflora, and diseases is also presented.

## 1 Introduction

The intestinal tract is the second “brain”, of human beings and is vital to human health. It is an important place for the absorption of nutrients and excretion of metabolic wastes in the body and contains more than 400 species of intestinal bacteria, which play an essential role in nutrient absorption, participating in body metabolism, and immune enhancement. The balance of intestinal microflora is very important to maintain human health. When the intestinal microflora is disturbed and out of balance, it can easily lead to a series of diseases, such as cancer, diarrhea, autism, and enteritis (1). Colorectal cancer is the second leading cause of cancer deaths in Europe, and in 2015 scholars from 10 European countries investigated the relationship between selenium and colorectal cancer incidence in up to 520,000 subjects, Results indicate that selenium levels is associated with the perceived level of colorectal cancer risk, which increased selenium intake in low selenium regions may decrease the risk as a result (2, 3). Meanwhile, experts from Penn State University found that selenium levels in within individuals were strongly associated with the development of inflammatory bowel disease, and that lower selenium levels were associated with greater susceptibility to inflammatory bowel disease. This is because selenium can be involved in regulating the intestinal inflammatory response, with changes in cellular oxidative status coupled with altered selenoprotein expression in macrophages driving the switch from a pro-inflammatory to an anti-inflammatory phenotype, resulting in the elimination of intestinal inflammation and restoration of the barrier role of the intestinal epithelium (4).

Se can be divided into organic and inorganic selenium. Cereals, meat, fish, and nuts are the best sources of Se. Among these, Brazil nuts, are one of the foods with the highest Se content, ranging from 1.80 to 320.80 μg Se/g (5). In addition, selenium intake is also regionally related, and studies have found that in areas with low soil selenium content, selenium levels in drinking water and crops are generally low, resulting in inadequate selenium intake by the local population (6). In China, 72% of the soil environment is deficient in selenium, which cannot meet the human body's demand for selenium (7). Therefore, scientific selenium supplementation is very important, usually you can supplement selenium-rich foods, in the case of serious selenium deficiency, you should supplement selenium-containing preparations. Sulfur amino acid analogs are organic forms of Se, including selenomethionine (SeMet), selenocysteine (SeCys), and methylated derivatives. The inorganic forms are selenates, such as selenate (SeO${}_{4}^{-2}$) and selenite (SeO${}_{3}^{-2}$). Indeed, the bioavailability of selenium depends mainly on its chemical form. Under normal conditions, organic selenium is absorbed more rapidly and is usually used in the biosynthesis of selenoproteins (8). The organic form of selenium (selenomethionine) has higher bioavailability and lower toxicity than its inorganic form (sodium selenite). In addition, selenium nanoparticles prepared from sodium selenite, ascorbic acid and chitosan, added to soy sauce, can enhance the antioxidant activity of soy sauce (9). Studies have shown that the biosynthesis of organic selenium can be accomplished by whole-cell biotransformation using sodium selenite under controlled *Bifidobacterium bifidum* BGN4 culture conditions, with a maximum organic selenium content of 207.5 μg/g in *Bifidobacterium selenium*-enriched BGN4 (10).

Organic or inorganic selenium is absorbed in the intestine and subsequently transported to the liver, where it is metabolized and produces selenoproteins, which are subsequently transported to other tissue sites in the body, thereby having an impact on human health. In addition, several lactic acid bacteria can maintain the sodium selenite state in the form of SeCys and SeMet in cells, thus providing a bioavailable form of Se, which is usually responsible for the poor absorption of inorganic selenium in human cells (11). The amount of Se intake has been reported to affect the barrier function and immune response in the intestine. Selenium is essential for maintaining the immune system, conversion of thyroid hormones, and reducing the risk of chronic diseases (12). In addition to this, selenium can balance intestinal microecology and avoid health damage caused by its imbalance. For example, under lead exposure, the gut experiences reduced microbiota diversity and oxidative stress, and the selenium-rich *Lactobacillus rhamnosus* SHA113 can effectively protect the gut from lead damage by forming an insoluble mixture with lead, which greatly facilitates the efficiency of lead excretion through the feces (13).

## 2 Selenium and human health

Selenium is one of the most important trace elements in the human body and has significant anti-inflammatory and antioxidant properties that exert antioxidant effects and protect the body from oxidative damage. Both deficiency and excess selenium are associated with the development of certain diseases. Insufficient serum levels of selenium may cause various diseases such as cardiovascular diseases (14, 15). On the other hand, excess selenium may lead to selenosis, which can cause symptoms such as fatigue, tachycardia, and diarrhea (16). Chronic selenium overdose can also lead to liver and kidney necrosis, neurological disorders, and damage to the reproductive and immune systems (5). A study of more than 13,000 followers over 12 years found that serum Se levels ≥135 μg/L was associated with reduced cancer mortality (17). Similarly, a systematic evaluation including 13 meta-analyses showed that high selenium levels were associated with lower morbidity and mortality from cardiovascular disease (18). In another study of over 40,000 randomized trial participants, selenium supplementation was found to reduce serum C-reactive protein levels and increase GPX levels, suggesting a positive effect of selenium on reducing inflammation and oxidative stress in cardiovascular disease (19).

In addition, Se and selenoproteins may play an important role in signaling pathways involved in the pathogenesis of certain diseases, such as IBD and cancer (20). Se deficiency and selenoprotein underexpression also impair innate and adaptive immune responses, and in the colon, Se deficiency and selenoprotein underexpression lead to an increase in inflammatory cytokines (21). Additionally, a low intake of Se may lead to a phenotype of gut microbiota more susceptible to colitis and *Salmonella typhi* infections (22). On the other hand, adequate or high levels of Se diet may optimize the intestinal microflora to prevent intestinal dysfunction and chronic diseases (22). Currently, there are more studies on the relationship between selenium and disease and fewer studies on selenium and intestinal microflora and selenium-intestinal microflora-human health.

## 3 Selenium and intestinal microflora

Approximately 100 trillion microorganisms, including bacteria, viruses, fungi, and protozoa, live in the human gut (23, 24). Gut microbes are capable of encoding more than three million genes and perform a range of metabolic processes that are impossible for humans, including the synthesis of vitamins and bioactive compounds, the synthesis of essential and non-essential amino acids, and the metabolism of indigestible carbohydrates, among other functions. In addition, intestinal bacteria play a role in nutrient absorption and act as a barrier to block pathogens. In this process, an important role is played by selenium, one of the essential elements responsible for DNA replication and transcription, and a key cofactor for antioxidant and cellular respiratory bacterial enzymes (25). For example, selenocysteine synthase (SelA) is a pyridoxal phosphate-dependent enzyme that catalyzes the formation of selenocysteine-tRNA in bacteria (26).

Selenium intake in food also affects the selenium status and expression of selenoproteins in the host. Intestinal microflora can use ingested Se to express their selenoproteins. Selenium binding protein 1 is involved in selenium metabolism and redox control and has been identified as a circulating biomarker of cardiac events in patients with the suspected acute coronary syndrome (27). Selenium also affects the composition and colonization of the intestinal microbiota and can have an impact on microflora diversity and composition (28). Dietary selenium can influence the colonization of gut microbes, which in turn can affect host selenium status and selenoprotein expression. Studies have shown that about 1/4 of bacteria have genes encoding selenoproteins, and some of them, such as *Escherichia coli, Clostridium difficile*, and *Enterobacteriaceae*, can colonize the human gastrointestinal tract (29).

The microbiota can influence selenium status, and although the human body and the gut microflora benefit from a symbiotic relationship, the two may compete for selenium when micronutrient (such as selenium) levels become limited (30). On the one hand, the gut microbiota promotes the biotransformation of selenium compounds. Besides, the uptake of selenoproteins by gut bacteria negatively affects the expression of selenoproteins in the host, but the adverse consequences of such effect on human health have not been demonstrated. Se metabolism in humans should be studied to assess whether to recommend an increase in Se intake (29). Furthermore, the effect of probiotics on Se is related to the composition and metabolism of the intestinal microbiota. Se is required for the conversion of T4–T3, and the gut microbiota can interact with Se to express its selenoproteins. Moreover, certain species of gut microbes can increase the bioavailability of selenium and prevent the toxicity produced by its excess (5). Some intestinal bacteria can prevent infection by selenium-dependent bacteria by competing for selenium or by producing metabolites that may be harmful to pathogenic bacteria. In the face of this type of bacterial infection, a complex interaction occurs between the host's immune response, the microbial pathogen, the microbiota, and the Se status of the host. Bacteria with Se-dependent enzymes can survive in the anaerobic conditions of the mammalian gut and these bacteria benefit from the host by using Se to increase their virulence and pathogenicity (31). It was shown that SeNPs with biomolecular shells synthesized by the probiotic bacterium *Lactobacillus casei* 393 have significant antioxidant and anticancer activities and could provide a better option for the synthesis of the biogenic element selenium with potential applications as anticancer and antioxidant agents (32).

It has been shown that anaerobic Trichoderma and Clostridium, particularly *Bacillus fumigatus*, are selenoprotein-rich prokaryotes. Evolutionary trends in Se and selenoprotein use suggest that there are more than 5,200 bacterial genomes, most of which are host-related, of which 2/3 do not use Se, indicating that this capacity has been lost over time (5, 33).

## 4 Selenium, intestinal bacteria and disease

A lack of Se can reduce an individual's immunity, allowing bacteria that do not require Se to survive to cause infection and lead to disease. The human gut microflora may also differ in the presence of Se, which can disrupt the balance of the human gut microflora by competing for Se or producing metabolites that may be harmful to disease-causing bacteria in order to prevent infection by Se-dependent bacteria (31). Crohn's disease and ulcerative colitis, collectively known as inflammatory bowel disease (IBD), are characterized by an imbalance in intestinal microbial ecology that leads to altered intestinal dynamics and secretion, allowing for visceral allergy and failure of gut-brain communication (34, 35). Selenium deficiency is common in IBD patients, up to 30.9% (36). Studies have shown that Se is important in improving IBD due to the ability of selenoproteins to reduce the inflammatory response (4). Furthermore, dietary Se content was positively correlated with the presence of Firmicutes and negatively correlated with warty colitis in IBD patients (37).

In addition to its effects on inflammatory bowel disease, selenium can also influence the gut microbiota to have an impact on thyroid function (38). The thyroid gland contains the highest levels of Se and several proteins involved in thyroid metabolism contain Se. The microbiota affects Se uptake and can alter the availability of L-thyroxine and the toxicity of propylthiouracil (PTU). In the presence of normal Se levels, the thyrotropin reductase system and SH-Px protect thyroid cells from damage by peroxides. A decrease in the number of lactic acid bacteria in patients with thyroid disease could confer the bioavailability of Se and its role in activating thyroid hormone conversion (39).

Se prevents oxidative damage during the synthesis of other hormones. In addition, the relationship between selenium and cancer affecting intestinal microflora cannot be ignored. Bacteria of the genus *Dorea* sp. are the most common species in the intestinal microbiota and their abundance is increased under Se deficient conditions, disrupting the homeostatic balance of the gut and affecting host metabolism and immunity, with consequent potential for diseases such as cancer, multiple sclerosis, and non-alcoholic liver disease (22, 40). Selenium deficiency and inadequate selenoprotein expression also impair innate and adaptive immune responses with high levels of inflammatory cytokines, especially at the colonic level. The impact of the gut microbiota on selenoproteins and other molecules related to redox homeostasis may have implications for the regulation of oxidative stress, apoptosis, inflammation, and immune responses, which appear to have a direct impact on cancer risk and development (41). In a human study, co-supplementation with probiotics containing *Lactobacillus acidophilus, B. bifidum, Bifidobacterium longum*, and selenium led to improvements in cognitive function and increased metabolic levels (42). Moreover, probiotics can reduce inflammatory factors and oxidative damage by producing short-chain fatty acids and reducing the production of free radicals in the intestine (5). Considering that selenium uptake by the intestinal microbiota occurs under unbalanced conditions, it may negatively affect the Se supply to the host, thus inducing cancer and intestinal dysfunction. The deficiency of selenoproteins and molecules related to redox homeostasis leads to a gut microbiota phenotype more susceptible to colitis, pathogenic infections, and cancer (22). Lower expression of different selenoproteins was found in colorectal adenoma and cancer tissues, while higher SELENOP concentrations were negatively associated with colorectal cancer risk (2). *Lactobacillus* is an important genus of bacteria in the human gut, capable of increasing Se concentrations in human cells, and the relative abundance of *S.butyricum* (*P* < 0.001) and significantly lower *Lactobacillus* (*P* < 0.001) was observed in thyroid adenomas (43).

## 5 Expectation

In conclusion, selenium deficiency may lead to phenotypic alterations in the gut microbiota, making humans more susceptible to the development of inflammatory bowel disease, thyroid disease, and cancer, among others. Despite some progress in research between selenium and gut bacteria, many questions remain to be addressed, such as what is the optimal selenium level for healthy gut microbiota? The link between gut microbiota, selenium status, and disease is still difficult to determine, and the complex interactions between microbiota, diet, and the human body may involve multiple mechanisms. Therefore, to address the above questions, a three-pronged approach should be taken.

1) Intestinal bacteria can absorb selenium from the human body, and the relationship between the optimal daily selenium intake in humans and the selenium requirement of normal intestinal microflora needs to be addressed. Selenium is mostly present in the human body in the form of selenocysteine, and the effects and differences of other forms of selenium on the balance of human intestinal microflora are worth exploring.
2) Although some clinical studies have revealed an association between gut microbiota composition and disease development, few studies have provided evidence for the direct role of Se in the gut microbiota. Most studies on selenium and gut bacteria have focused on mice, few have been conducted on humans. Therefore, the Se-gut microbiota-disease relationship needs to be explored in humans, and more clinical studies are recommended.
3) The vast majority of intestinal microflora is not culturable, which poses some difficulties for selenium-gut microflora-disease studies. Based on high-throughput sequencing and the multi-omics combination can exactly bridge the above shortcomings.

## Author contributions

JC and HZ conceived and wrote the first draft of the manuscript. JC, WS, XC, and HZ revised each part of the manuscript in detail. All authors participated in the revision of the manuscript, read, and approved the submitted version.
